# Native Chilean Berries Preservation and In Vitro Studies of a Polyphenol Highly Antioxidant Extract from Maqui as a Potential Agent against Inflammatory Diseases

**DOI:** 10.3390/antiox10060843

**Published:** 2021-05-25

**Authors:** Tamara Ortiz, Federico Argüelles-Arias, Belén Begines, Josefa-María García-Montes, Alejandra Pereira, Montserrat Victoriano, Victoria Vázquez-Román, Juan Luis Pérez Bernal, Raquel M. Callejón, Manuel De-Miguel, Ana Alcudia

**Affiliations:** 1 Departamento de Citología e Histología Normal y Patológica, Universidad de Sevilla, Avda. Sánchez-Pizjuán s/n, 41009 Sevilla, Spain; tamara.ortiz.cerda@gmail.com (T.O.); mvazquez2@us.es (V.V.-R.); 2 Departamento de Medicina, Universidad de Sevilla, Avda. Sánchez-Pizjuán s/n, 41009 Sevilla, Spain; farguelles1@us.es (F.A.-A.); jfgarcia@us.es (J.-M.G.-M.); 3 Departamento de Gastroenterología, Hospital Universitario Virgen Macarena, c/Dr. Fedriani nº 3, 41009 Sevilla, Spain; 4 Departamento de Química Orgánica y Farmacéutica, Universidad de Sevilla, c/Prof García González nº 2, 41012 Sevilla, Spain; bbegines@us.es; 5 Departamento de Nutrición y Dietética, Escuela de Ciencias de la Salud, Universidad de Desarrollo Concepción Barrios Arana1735, Concepción 4070146, Chile; alejandra.pereira@udd.cl; 6 Departamento de Nutricion y Dietetica, Facultad de Farmacia, Universidad de Concepción, Concepción, Chile. Barrio Universitario s/n, Concepción 4070146, Chile; mvictoriano@udec.cl; 7 Departamento de Química Analítica, Universidad de Sevilla, c/Prof García González nº 2, 41012 Sevilla, Spain; juanluis@us.es; 8 Departamento de Nutrición y Bromatología, Toxicología y Medicina Legal, Universidad de Sevilla, c/Prof García González nº 2, 41012 Sevilla, Spain; rcallejon@us.es

**Keywords:** maqui berry extract, preservation methods, antioxidant activity, polyphenols and anthocyanins content, oxidative stress, inflammation, RAW 264.7 cells, HT-29 cells, inflammatory bowel disease

## Abstract

The best conservation method for native Chilean berries has been investigated in combination with an implemented large-scale extract of maqui berry, rich in total polyphenols and anthocyanin to be tested in intestinal epithelial and immune cells. The methanolic extract was obtained from lyophilized and analyzed maqui berries using Folin–Ciocalteu to quantify the total polyphenol content, as well as 2,2-diphenyl-1-picrylhydrazyl (DPPH), ferric reducing antioxidant power (FRAP), and oxygen radical absorbance capacity (ORAC) to measure the antioxidant capacity. Determination of maqui’s anthocyanins profile was performed by ultra-high-performance liquid chromatography (UHPLC-MS/MS). Viability, cytotoxicity, and percent oxidation in epithelial colon cells (HT-29) and macrophages cells (RAW 264.7) were evaluated. In conclusion, preservation studies confirmed that the maqui properties and composition in fresh or frozen conditions are preserved and a more efficient and convenient extraction methodology was achieved. In vitro studies of epithelial cells have shown that this extract has a powerful antioxidant strength exhibiting a dose-dependent behavior. When lipopolysaccharide (LPS)-macrophages were activated, noncytotoxic effects were observed, and a relationship between oxidative stress and inflammation response was demonstrated. The maqui extract along with 5-aminosalicylic acid (5-ASA) have a synergistic effect. All of the compiled data pointed out to the use of this extract as a potential nutraceutical agent with physiological benefits for the treatment of inflammatory bowel disease (IBD).

## 1. Introduction

Inflammatory bowel disease (IBD) includes a group of diverse inflammatory diseases with chronic relapsing disorders and uncontrolled inflammation of the gastrointestinal tract such as ulcerative colitis (UC) or Crohn’s disease (CD). Additionally, the prevalence and incidence of IBD in adults and children has increased worldwide during the last decades, mainly in Westernized countries of Europe and North America, and more recently in industrialized countries. In Europe, more than 2.2 million patients with IBD have been reported and it is increasing more and more in the last decade [1,2]. Unfortunately, no clear or definitive evidence has been found related to the pathogenesis’ origin mainly due to its complexity. Urban populations, lifestyle, stress, smoking, and non-healthy diets based on high intakes of fats and poor intakes in vegetables or fruits are associated with alteration of intestinal microbiota that increase the risk of IBD in genetically susceptible individuals [3,4]. On the other hand, physiological concentrations of reactive oxygen species (ROS) are essential for cell survival and several physiological processes, including protein phosphorylation, activation of transcription factors, cell differentiation, apoptosis and cell immunity, etc. [5]. The effect of oxidative stress (OS) may have an impact on the pathophysiology of numerous chronic diseases, by reactions with the fatty acids of membranes, proteins, and DNA damage [6]. At the level of the gastrointestinal tract, OS can result in harmful effects such as stimulation of leukocytes infiltration, epithelial damage, mucus content depletion, and rupture of colonic barriers, along with inflammatory mediator release including inflammatory cytokines and arachidonic acid metabolites, as well as ROS, leading to oxidative damage and contributing to the development of IBD [7].

In this context, nutraceuticals or functional foods have gained popularity worldwide due to their high-value bioactive compounds [8]. Fruits and especially edible berries are rich sources of a wide variety of antioxidant phenols and include a high content of polyphenols and flavonoids [9]. In general, berries exhibit antioxidant and anti-inflammatory properties, decreasing OS and increasing intestinal protection, and can promote the prolongation of the remission phase and the duration/intensity of acute phases [10,11]. Among the most interesting berries for their high antioxidant capacity (AC) are the maqui and murta [12]. *Aristotelia chilensis* (Molina) Stuntz or maqui, endemic from Chile and widely consumed by its inhabitants, is an indigenous plant used as medicine. It contains an exceptional contribution both in concentration and in a variety of polyphenols (anthocyanins and non-anthocyanins) [13], with an extraordinary antioxidant power and a remarkable anti-inflammatory effect [14]. Moreover, *Ugni molinae*, known as murtilla or murta, is a wild berry native plant occurring in the lowlands of the southern mountains of Chile [15] and it is an interesting berry to be compared to maqui. A study reported by Speisky et al. [16] showed that the AC of maqui is still much higher than other cultivated berries and natural products. In general, berries are commonly consumed not only in fresh and frozen forms but also as processed tablets or pills [9]. For this reason, several studies have evaluated the effects and impact of different methods of food preservation on the maintenance of bioactive compounds and antioxidant activity. For example, the thermal effect caused by the different sterilization methods (thermal, microwave, and ultrasonic processing) on canned berries decreases the antioxidant activity and total content of anthocyanins and phenolics, whereas a low temperature storage better preserves the bioactive compounds and antioxidant activity [17,18]. Once the fruit has been preserved to retain its best characteristics as an antioxidant, an extraction to separate the desired natural products from the raw materials is needed. In this sense, and according to the highest antioxidant power of the maqui berry, various ways of production of maqui extracts have been described aiming to preserve not only the content of bioactive compounds but also the highest antioxidant activity [19,20]. Among them, the methanolic extract method has shown to preserve the highest AC, measured by oxygen radical absorbance capacity (ORAC), the radical scavenging activity using 2,2-diphenyl-1-picrylhydrazyl (DPPH) [21], ferric reducing antioxidant power (FRAP) [22], and total polyphenols content (TPC), measured by the Folin-Ciocalteu method [21]. In total, three groups of polyphenolic compounds have been described: Anthocyanins (delphinidin 3-*O*-sambubioside-5-*O*-glucoside, delphinidin 3,5-*O*-diglucoside, cyaniding 3-*O*-sambubioside-5-*O*-glucoside, cyaniding 3,5-*O*-diglucoside, delphinidin 3-*O*-sambubioside, delphinidin 3-*O*-glucoside, cyaniding 3-*O*-glucoside, and cyaniding 3-*O*-sambubioside), flavonols (quercetin, myricetin, kaemphenol, and its derivates), and phenolic acids (gallic acid). Recent studies have shown that the most abundant compounds are delphinidin and cyanidin derivatives that confer possible health benefits such as anti-inflammatory and antioxidant effects [23,24]. The characterization of the anthocyanin profile in maqui fruit has been performed using high-performance liquid chromatography-diode array detection and mass spectroscopy (HPLC-DAD-MS) [25], with delphinidin-3-sambubioside-5-glucoside as the majority delphinidin derivative, accounting for 34% of the total anthocyanidin level in maqui [26,27].

Interestingly, different authors have described a beneficial biological effect of maqui extract in cellular culture. For example, pre-incubation of fibroblast and Caco-2 cells with anthocyanin rich maqui berry extract has shown a protective effect against OS along with a high capacity to inhibit human low-density lipoprotein (LDL) oxidation [28]. Additionally, the maqui extract exhibits an important anti-inflammatory effect in RAW 264.7 macrophages cells [29] by inhibition of gene expression of *iNOS, COX-2, TNF-α, IL-6*, and *IL-10* [24,30].

The aim of the present study is to estimate the TPC and evaluate the AC of two native Chilean berries in different types of preservation methods: Fresh, refrigerated, and frozen. Furthermore, the correlations between the phenolic content and antioxidative activity were evaluated. To obtain a large-scale amount of maqui berry extract, richer in polyphenols content and with a more important antioxidant capacity than murta was an objective, together with exploring the curative benefits as a potential agent in the treatment of IBD. The maqui extract was evaluated in vitro, testing its viability, cytotoxicity, and antioxidant properties in colonic epithelial cell lines (HT-29) and macrophages (RAW 264.7).

## 2. Materials and Methods

### 2.1. Reagents and Apparatus

Chemical reagents and solvents for the extraction (MeOH, HCl), total polyphenols content analysis (Folin−Ciocalteu reagent, gallic acid, and sodium carbonate), DPPH assay, 2,2′-azobis(2-amidinopropane) dihydrochloride (AAPH) and reagents for FRAP analysis (sodium acetate trihydrate/glacial acetic acid, Trolox (6-hydroxy-2,5,7,8-tetramethylchroman-2-carboxylic acid), TPTZ (2,4,6-tripyridyl-s-triazine), and iron(III) sulphate (Fe_2_(SO_4_)_3_·7H_2_O)) were purchased from Sigma-Aldrich (Saint Louis, MO, USA) and used without further purification. Determination of maqui’s anthocyanins was performed by ultra-high-performance liquid chromatography (UHPLC-MS/MS) coupled to a hybrid quadrupole-orbitrap mass spectrometry system (Qexactive, Thermo Fisher, Waltham, MA, USA) with electrospray ionization (HESI-II). Separation was carried out using an Acquity UPLC BEH C18 (2.1 × 100 mm i.d., 1.7 um particle size) column (Waters, Milford, MA, United States) set to 40 °C. Celite^®^ Hyflo Supercel and membrane filter (filter paper, MFS) for filtration were from Merk (Darmstadt, Germany) and Biorad (California, USA), respectively. Fetal bovine serum (FBS), trypsin/ethylenediaminetetraacetic acid (EDTA), penicillin/streptomycin (P/S), McCoy’s 5a, and dulbecco’s modified eagle medium (DMEM) high glucose culture medium were purchased from Biowest (Nuaillé, France). *N*-acetyl-l-cysteine 5 (NAC), 5-aminosalicylic acid (5-ASA), and lipopolysaccharide (LPS) were obtained from Sigma-Aldrich (Saint Louis, MO, USA). The Alamar Blue^®^ cell viability reagent and 2′,7′–dichlorofluorescin diacetate (DCFDA) were purchased from Thermo Fisher Scientific (Waltham, MA, USA). The ultrasound, rotary evaporator with controlled heating, lyophilizer, optical microscope, and microplate reader were purchased from Hielscher Ultrasound Technology (UP400S, Wanaque, NJ, USA), Telstar Cryodos (Josep Tapiolas, Terrassa, Spain), Leica (Wetzlar, Germany) and Tecan (Männedorf, canton of Zürich, Switzerland) companies, respectively.

### 2.2. Plant Material and Preservation Methods

Wild maqui and murta berries have been collected from areas near the Andes Mountains: Bio-Bio and Araucanía Region, Chile. The fruits were separated in three groups for analytical procedures: (a) Refrigeration at 4 °C for 1 week, (b) freezing at −18 °C for 1 month, and (c) fresh fruits. The fresh samples were analyzed immediately and treated according to the procedure described by Rubilar et al. [12]. All of the berries were dried at 35 °C at an equative weight. Subsequently, grinding and sifting steps were conducted. The samples were macerated with ethanol (50% *v*/*v* in water, 20% *w*/*v* at room temperature for 2 h). The extracts were filtered through a membrane filter, centrifuged at 2600 relative centrifugal force (RCF) for 30 min at 20 °C, and lyophilized.

### 2.3. Preparation of Polyphenolic Maqui Extract (Ach)

**Figure 1:**
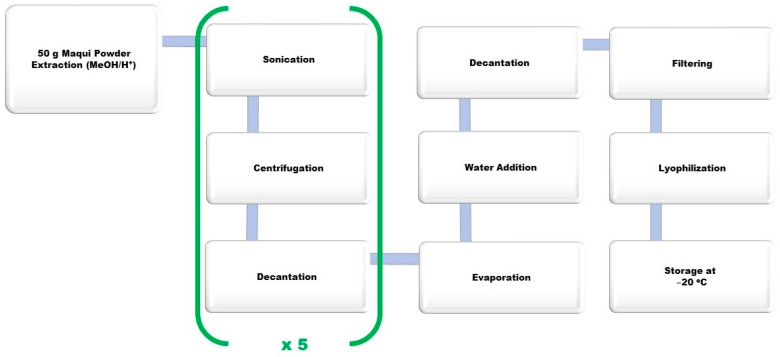
Conceptual scheme of maqui extraction procedure for 50 g.

For the large-scale extract, lyophilized wild maqui was used including seeds, skin, and pulp obtained from a powder wild harvested in Patagonia, marketed by “ISLA NATURA DE CHILE^®^” (Los Lagos, Chile). In order to quantitatively extract the raw maqui polyphenols, the extraction procedure was carried out using acidic methanol (MeOH/H^+^) following a method published by Genskowsky et al. [20], although some modifications were implemented (Figure 1). The entire procedure was performed in darkness and at room temperature as described by Miranda Rottmann et al. [28] to avoid decomposition processes. A suspension of lyophilized maqui powder (50 g) in 0.1% HCl in MeOH (250 mL) was stirred for 15 min. Afterwards, the mixture was sonicated with an ultrasound device at maximum power for 2 min. The sample was centrifuged at 2600 RCF for 10 min and allowed to precipitate for 5 min. Finally, the supernatant was recovered in an Erlenmeyer (Thermo Fisher Scientific, Waltham, MA, USA). The previous process was repeated 5 times identically. Once all the supernatants were compiled, the solution was placed in a rotary evaporator for 2–3 h at 35 °C, in order to evaporate the organic solvent and concentrate the extract. The extract was resuspended in pure water, centrifuged at 2600 RCF for 4 min, and the supernatant recovered. Then, the filtering process was performed using a vacuum, with a short pad of celite, then passed through filter paper #1 (porosity 100–150 MM) and then #3 (porosity 40–100 MM). Finally, the lyophilization process was carried out for 24 h to prevent enzymatic and chemical changes, protein denaturation, loss of aromas, and easily oxidizable components, to get a highly hydroscopic powder that was stored at −20 °C.

### 2.4. The 2,2-Diphenyl-1-Picrylhydrazyl (DPPH) Free Radical Scavenging Activity Assay

This assay is based on the decoloration of DPPH free radical solution due to the free radical scavenging effect of antioxidants. The radical scavenging activity was estimated by the method described by Brand Williams [31] with minor modifications: 800 μL of extracts were mixed with 3.9 mL of methanolic solution containing 0.1 mmol of radical DPPH. After 30 min of incubation in a dark chamber, absorbance was measured at 520 nm. Results were expressed as the half maximal effective concentration EC50, defined as the concentration that causes a 50% decrease in the initial DPPH concentration.

### 2.5. Oxygen Radical Absorbance Capacity (ORAC) Assay

The ORAC assay was performed according to the method described by Zheng and Wang [32]. This assay estimates the ability of antioxidant components to inhibit the decline of oxidative degradation of a fluorescent molecule ((*R*)-phycoerythrin (*R-*PE)) induced by peroxyl radical generator AAPH. The assay was prepared by mixing 1.7 mL of phosphate buffer (pH 7.0), 100 μL of *R*-PE (3.4 mg/L), 100 μL of AAPH, and 100 μL of each sample. Samples without radicals were preincubated at 37 °C for 15 min. Fluorescence was recorded every 5 min at 540 nm excitation until the last fluorescence reading decreased 5% from the first reading. The antioxidant activity was expressed as mg of Trolox equivalents (TE) per gram of dry mass (DM) of plant material used for extraction (mg TE/g DM), according to the standard curve previously prepared.

### 2.6. Ferric Reducing Antioxidant Power Estimation (FRAP) Assay

AC was determined by FRAP, according to Benzie and Strain [33] with some modifications. The FRAP assay is a simple, reproducible, rapid, and inexpensive method that measures the reductive ability of antiradical species and is evaluated by the transformation of ferric ion Fe^3+^ to ferrous Fe^2+^. This last one gives a blue complex by the reaction with TPTZ and is compared against the AC of Trolox as a measure of total antioxidant capacity. The FRAP reagent was prepared by mixing in this order, acetate buffer (0.3 M, pH 3.6), 10 mM TPTZ in 40 mM HCl, and 20 mM Fe_2_(SO_4_)_3_·7H_2_O. A fresh acetate buffer was previously prepared by dissolving 3.1 g of sodium acetate trihydrate and 16 mL of acetic acid in 1 L of distilled water. The ready-to-use FRAP reagent was also freshly prepared by mixing the acetate buffer, TPTZ, and Fe_2_(SO_4_)_3_·7H_2_O in 10:1:1 proportion. Two solutions at different concentrations of extract were made from a stock solution of 1.28 mg/mL to determine its antioxidant activity. In summary, 50 µL of extracts were added to the diluted FRAP reagent in MeOH (1 mL FRAP reagent mixed with 2 mL MeOH). The measure of absorbance was recorded at 593 nm after 30 min in darkness at room temperature against a blank (FRAP diluted reagent previously prepared without the extract). The standard curve was prepared using different concentrations of Trolox and the results were expressed as μmol TE/100 g.

### 2.7. Determination of Total Polyphenols Content (TPC)

The Folin–Ciocalteu method is a type of assay based on an electron transfer mechanism. To provide basic conditions, Na_2_CO_3_ is used in the solution to dissociate phenolic protons that yield phenolate anions. Phenolate anions can reduce the Folin–Ciocalteu reagent through the reduction of Mo^6+^ to Mo^5+^ that results in a blue chromophore constituted by a phosphotungstic-phosphomolybdenum complex that can be measured spectrophotometrically [34]. The following methodology described by Slinkard and Singleton [35] was used for measurement. Then, 20 µL of the sample, 1580 μL distilled water, 100 μL Folin–Ciocalteu reagent, and 300 μL Na_2_CO_3_ (200 g/L) were added to a glass tube. These solutions were mixed and incubated at 40 °C for 30 min in a water bath. Absorbance was measured at 740 nm against a blank solution. Gallic acid was used as a standard. All results were expressed as milligrams of gallic acid equivalent (GAE) per gram of dry matter (DM).

### 2.8. Determination of Anthocyanin Profile by UHPLC-HRMS/MS

Determination of maqui’s anthocyanins was performed with a Thermo Scientific Liquid Chromatography system consisting of a binary UHPLC Dionex Ultimate 3000 RS connected to a quadrupole-orbitrap Qexactive hybrid mass spectrometer (Thermo Fisher Scientific, Waltham, MA, USA) with a heated electrospray ionization probe (HESI-II). The Xcalibur software (Thermo Fisher Scientific, Waltham, MA, USA) was used for instrument control and data acquisition. Separation was carried out using an Acquity ethylene bridged hybrid (BEH) C18 (2.1 × 100 mm, 1.7 µm particle size) column (Waters, Milford, MA, USA) set to 35 °C at a flow rate of 0.4 mL/min. A binary gradient consisting of (A) water/formic acid 95:5 (*v*/*v*) and (B) acetonitrile/formic acid 95:5 (*v*/*v*) was used with the following elution profile: 0–2 min 5% B, 2–12 min from 5% to 100% B, 12–13 min 100% B, and 13–15 min 5% B. The injected volume was 5 μL. The lyophilized extract was dissolved in 1 mL of mobile phase A and microfiltered with a 0.2 µm nylon filter. A Data Dependent Acquisition method (TOP5) was used in a positive mode at a resolution of 70,000 and 17,500 at *m*/*z* 200 full-width half-maximum (FWHM) for Full Scan and Product Ion Scan, respectively. HESI source parameters were: Spray voltage, 3.5 kV; S lens level, 50; capillary temperature, 320 °C; sheath, auxiliary, and sweep gas flow, 50, 13, and 3, respectively (arbitrary units); and probe heater temperature, 425 °C. For data treatment, the TraceFinder 5.1. software (Thermo Fisher Scientific, Waltham, MA, USA) was used. The identification was made by comparing (maximum deviation of 5 ppm) the exact masses of the pseudomolecular ion and their fragment ions with the data contained in an anthocyanins database with 12 possible compounds. The retention time of standard compounds and isotopic pattern scores higher than 80% were also required. The following anthocyanin standards were used for identification purposes: Cyanidin-3-glucoside, cyanidin-3-galactoside, malvidin-3-glucoside, peonidin-3-glucoside, and delphinidin-3-glucoside. Anthocyanin compounds were quantified using the areas of the aglycone counterparts.

### 2.9. Cell Culture

Human colorectal adenocarcinoma, epithelial, adherent (HT-29 cells) provided by the Department of Pharmacology (Seville University, Seville, Spain), were cultured in McCoy’s 5a culture medium, and murine macrophage cells, adherent (RAW 264.7 cells) were obtained from Centro Andaluz de Biología Molecular y Medicina Regenerativa (CABIMER, Seville, Spain) and cultured in DMEM high glucose. Both cell lines were supplemented with 10% FBS 1% P/S and incubated at 37 °C and 5% CO_2_, with the medium changing every 2 days. Cells were maintained in 75 cm^2^ flasks and, when they reached 80–90% confluence in the flask, were harvested using trypsin/EDTA (0.5% *v*/*v*).

### 2.10. Viability and Cytotoxic Assays

Cell viability was determined using the Alamar Blue^®^ reagent (Invitrogen, Carlsbad, CA, USA) based on the quantitative colorimetric assay following the manufacturer’s protocols. For all experiments, HT-29 and RAW 264.7 cells were seeded in 24-well plates (5 × 105 cells/well) and incubated for 24 h to allow the cells to adhere to the plate surface. Ach was prepared in distilled H_2_O and diluted in a cell culture medium. The concentrations of Ach in the in vitro experiments were based on data previously published on fruit extract of other berries [36,37]. Thus, treating the cells with 100, 200, and 300 μg/mL of Ach was chosen. HT-29 cells were exposed to H_2_O_2_ at 0.05% and incubated with or without Ach at different concentrations during 24 or 48 h. NAC, a known antioxidant, was used as a control. Cell viability of LPS (1.0 µg/mL)-induced RAW 264.7 cells was tested to determine the potential cytotoxic effect of increasing concentrations of Ach for 12 h. The 5-aminosalicylic acid (5-ASA), an anti-inflammatory drug, was used as a control according to a previously published work [38]. The culture supernatants were collected, and the absorbance of each one was measured at 570 nm using a microplate reader. The cell viability was calculated as follows:

(1) $$Cell\;viability\;\left(\%\right)=\frac{Abs\;\left(experiment\right)-Abs\;\left(blank\right)}{Abs\;\left(control\right)-Abs\;\left(blank\right)}\times 100$$

### 2.11. Determination of Reactive Oxygen Species (ROS) by the DCFH-DA Assay

A fluorescent 2′,7′-dichlorofluorescein diacetate (DCFH-DA) assay was performed to determine the intracellular ROS concentrations according to the method reported by Miranda-Rottmann [28] with modifications. Cells were seeded at 1 × 10^5^ cells/well in 96-well plates in a final volume of 100 µL of culture medium per well. When the cells reached a confluence of 80%, HT-29 and RAW 264.7 cells were incubated with H_2_O_2_ and LPS, respectively in the presence or absence of Ach and internal control (NAC and 5-ASA) for 1 h with 5% CO_2_ at 37 °C. After removing the medium, cells were washed twice with 50 µL/well of PBS, incubated at 37 °C for 30 min with 25 μM DCFH-DA, and stored under at −20 °C. For the DCFH-DA assay, a culture medium was used without phenol red and without supplementation to avoid interference with fluorescence emission. The fluorescence was measured at Ex/Em: 485/530 nm using a fluorescence microplate reader.

(2) $$Oxidation\;\left(\%\right)=\frac{Fluorescence\;\left(experiment\right)-Fluorescence\;\left(w/o\;cells\right)\;}{Fluorescence\;\left(control\right)-Fluorescence\;\left(w/o\;cells\right)}\times 100\;$$

### 2.12. Statistical Analysis

The results were expressed as the mean ± standard error of the mean (SEM) and ± the standard deviation (SD) according to the particular characteristics of the tests. All experiments were replicated three times. Heterogeneity was tested using Levene’s test and the Shapiro-Wilk test for normality. The continuous variables that are normally distributed were compared through the parametric Student *t*-test (two sample *t*-test) and proportions were tested using the two samples test calculator (prtesti). The analysis of differences between groups was evaluated by one-way analysis of variance (ANOVA) tests when the variables were normally distributed, followed by Bonferroni’s post-hoc test. Descriptive statistics and tests were performed at a significance level of 0.05 using the STATA software (version 12, 2011, StataCorp, College Station, TX, USA).

## 3. Results

### 3.1. Antioxidant Capacity (AC) by DPPH and ORAC of Maqui and Murta in Different Preservation Methods

The results of the analysis of the AC of maqui and murta, as estimated by the free radical scavenging through the DPPH method and ORAC assay, are shown in Table 1. Through the DPPH assay it is observed that the AC of maqui, regardless of all different types of preservation methods, is four times higher than murta. In the same way, the ORAC values indicate that maqui has a significantly higher AC than murta in fresh, refrigerated, and frozen samples. When AC of maqui and murta was estimated by these methods, results showed that both berries have greater AC in fresh and frozen storage, in comparison with the refrigerated state.

### 3.2. Total Polyphenols Content (TPC) of Maqui and Murta in Different Preservation Methods

The TPC values for maqui and murta estimated by the Folin-Ciocalteu method are shown in Table 2. It can be observed that maqui, with all the preservation methods, showed a significantly higher polyphenol content than murta. The TPC data from refrigerated samples presented the lowest values in comparison with fresh and frozen samples, showing statistical significance. On the other hand, when the data of maqui and murta were compared in fresh versus frozen state, the values were very similar.

### 3.3. Total Polyphenols Content (TPC) and Antioxidant Capacity (AC) of Polyphenolic Maqui Extract (Ach)

The most important factors that affect the polyphenol content in fruits are usually genotype, environment, geographical location, and altitude, as well as season, soil, maturity stage at harvest, and post-harvest conditions [20]. The Folin-Ciocalteu method is the most widely used assay for the estimation of TPC in a wide range of samples, including fruits, juices, and wine [39,40]. The results of the Folin-Ciocalteu method show that the lyophilized extracts of the maqui fruit content have an average TPC of 39.02 mg/g of polyphenols expressed as gallic acid. The results regarding the AC of the maqui extract to reduce Fe^3+^ to Fe^2+^, indicate that the Ach has an AC of 69.48 mmol Trolox/100 g sample.

### 3.4. Characterization of Extracts

**Figure 2:**
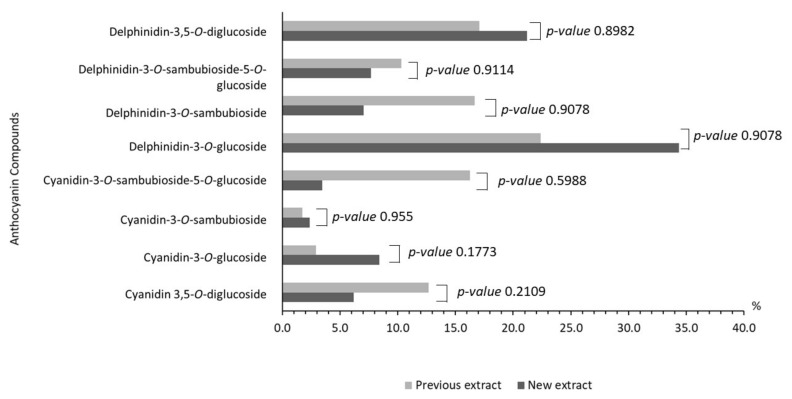
Determination of individual anthocyanin compounds in maqui extract by ultra-high-performance liquid chromatography (UHPLC-MS/MS) Orbitrap. Our data (new extract; dark gray) are presented according to the percentage of anthocyanin compound and compared with the anthocyanins profile previously reported (previous extract; light gray) [20].

Characterization of the maqui’s anthocyanins extract was performed by the UHPLC-MS/MS with an electrospray ionization that enables comparison of *m*/*z* signals and fragment ions of the anthocyanin pattern. Regarding the maqui composition, delphinidin-3-*O*-glucoside was the major component (*m/z*: 465.1, 34.3%), followed by delphinidin-3,5-*O*-diglucoside (*m/z*: 627.1, 21.2%), cyanidin-3-*O*-glucoside (*m/z*: 449.1, 8.3%), delphinidin-3-*O*-sambubioside-5-*O*-glucoside (*m/z*: 759.1, 7.7%), delphinidin-3-*O*-sambubioside (*m/z*: 597.1, 7.0%), cyanidin-3,5-*O*-diglucoside (*m/z*: 611.1, 6.2%), cyanidin-3-*O*-sambubioside-5-*O*-glucoside (*m/z*: 743.2, 3.4%), cyanidin-3-*O*-sambubioside (*m/z*: 581.1, 2.3%), as main components of the extraction procedure performed. Other similar structures identified were malvidin-3-*O*-glucoside (*m/z*: 493.1, 0.7%), pelargonidin-3-*O*-glucoside (*m/z*: 433.1, 0.1%), and peonidin-3-*O*-glucoside (*m/z*: 463.1, 0.05%). The anthocyanin profile is shown in Figure 2 and is compared with a previously reported one [20], without finding statistical differences.

### 3.5. Viability and Cytotoxicity

**Figure 3:**
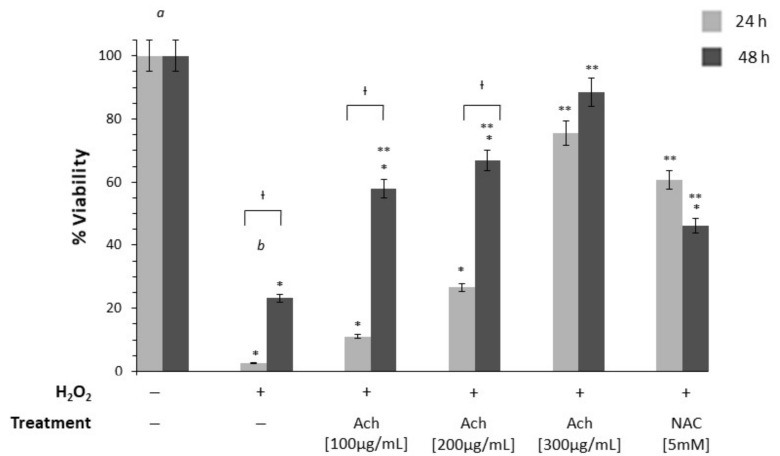
Ach presents a protective effect on the viability of HT-29 cells exposed to 0.05% H_2_O_2_ and treated during 24 and 48 h with different concentrations of Ach. The viability of cells without H_2_O_2_ was 100%. Data are expressed as the mean ± SEM. A *p*-value < 0.01 was considered statistically significant: * With respect to *a*, ** with respect to *b*, Ɨ between 24 and 48 h. Positive sign (+): presence of treatment; negative sign (−): absence of treatment.

**Figure 4:**
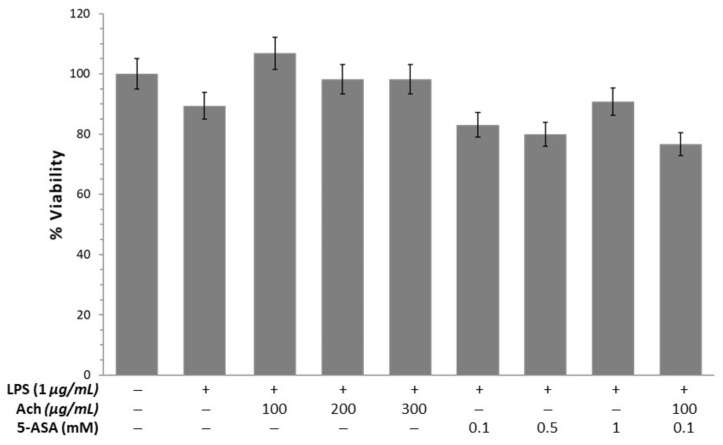
Ach shows no cytotoxicity effects in RAW 264.7 cells treated with lipopolysaccharide (LPS) (1.0 µg/mL). Data are expressed as the mean ± SEM. The viability of cells without LPS was 100%. Positive sign (+): presence of treatment; negative sign (−): absence of treatment.

To test the protective effect of maqui, HT-29 cells were incubated with increasing concentrations of Ach for 24 and 48 h and compared to NAC (Figure 3). Initially, the effect of H_2_O_2_ on epithelial cells was determined revealing a significant reduction (*p*-value < 0.01) of the viability. When HT-29 cells were incubated with Ach the results showed that all concentrations (100, 200, and 300 μg/mL) tested caused a significant increase of viability at 24 and 48 h. The effect of Ach on cell viability was higher than that of 5 mM NAC. To examine the cytotoxic effects of Ach, the viability of RAW 264.7 macrophages was evaluated at concentrations of 100, 200, and 300 μg/mL of Ach for 24 h. As shown in Figure 4, no notable effects on cell viability in LPS-stimulated RAW 264.7 cells were observed with or without treatment. When comparing Ach with different concentrations of 5-ASA (0.1, 0.5, and 1 mM) in the presence of LPS, no significant cytotoxicity was found, even when mixing 5-ASA (0.1 mM) and Ach (100 μM/mL).

### 3.6. Oxidative Stress

**Figure 5:**
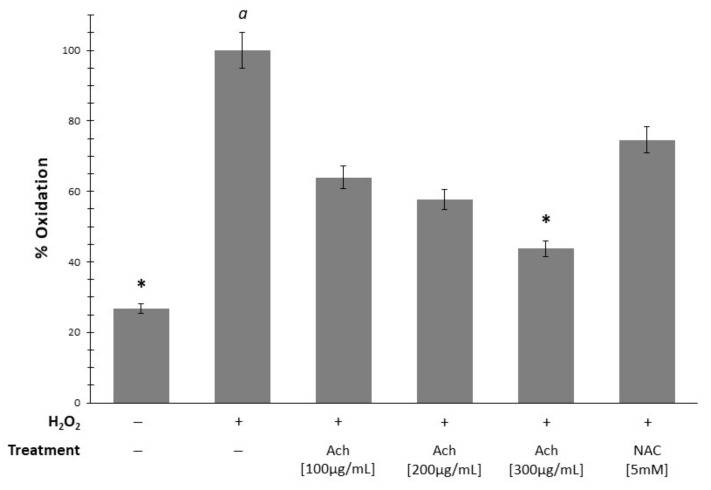
Supplementation with Ach to HT-29 cells stressed with 0.05% H_2_O_2_ decreases oxidation status in a dose-dependent manner, and more efficiently than NAC. Data are expressed as the mean ± SEM and the measured percent oxidation were expressed as a percentage from the positive control (cells induced by H_2_O_2_). *A *p*-value < 0.05 indicates significant differences with respect to *a*. Positive sign (+): presence of treatment; negative sign (−): absence of treatment.

**Figure 6:**
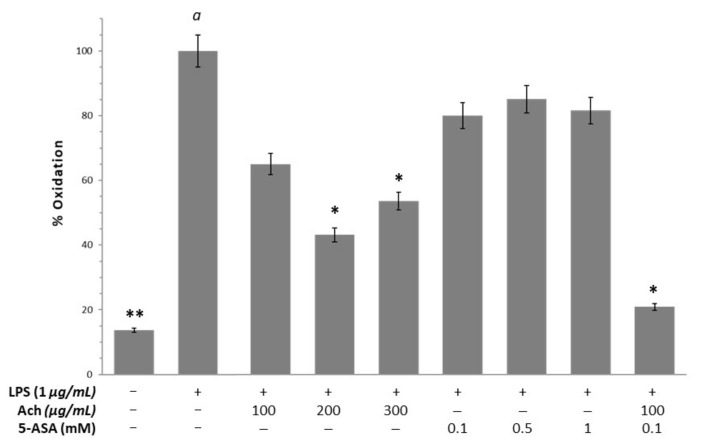
Ach inhibits oxidative stress in RAW 264.7 macrophages stimulated with LPS (1.0 µg/mL). The cells incubated with Ach (100 μg/mL) plus 5-aminosalicylic acid (5-ASA) (5 mM) presented the lowest percentage of oxidation. Data are expressed as the mean ± SEM and the measured percent oxidation were expressed as a percentage from the positive control (cells induced by H2O2). * A *p*-value < 0.05 and ** *p*-value < 0.01 indicate significant differences with respect to *a*. Positive sign (+): presence of treatment; negative sign (−): absence of treatment.

Figure 5 shows a significant increase of oxidation in HT-29 cells when H_2_O_2_ (*p*-value < 0.05) was added. These results were inhibited by all concentrations of Ach in a dose-dependent way, reaching significance at 300 μg/mL. In parallel, when epithelial cells exposed to H_2_O_2_ were treated with NAC, it was possible to observe a decrease in oxidation, although not significant. In RAW 264.6 macrophages, LPS stimulation led to the increment of oxidation, demonstrating the relationship between inflammation and OS (Figure 6). Ach effectively suppressed the LPS-induced oxidation in a dose-dependent manner, showing a significant reduction with the highest doses (200 and 300 μg/mL). At the same time, these results indicated that the suppression of oxidation could not be attributable to the direct cytotoxic effect of Ach. Of interest is the fact that the 0.1 mM of 5-ASA plus 300 μg/mL of Ach concentration had an inhibition effect comparable to the control conditions (cells in culture medium without LPS), achieving values of basal oxidative state.

## 4. Discussion

The polyphenolic content in several berries has been widely proved to be the major contributor to AC [39,41] and the way of consumption (fresh, processed or stored for long-term) could be relevant to preserve polyphenol compounds and its beneficial effects, including other phytochemicals that also play a critical role. In the present work, it was demonstrated that maqui has greater AC than murta, and significant differences of TCP in fresh fruit values, with 75.3 mg GAE/g FW for maqui and 48.0 mg GAE/g FW for murta. These findings are in agreement with Salvia-Trujillo et al. [42] who reported a depletion of antioxidant “Vitamin C” during refrigerated storage, which was correlated with AC. Additionally, other studies reported that polyphenols from crude extracts of maqui were also much higher than crude extracts from murta [12], and that maqui fruit does not decrease polyphenol and anthocyanin contents during desiccation, cooling or freezing processes [43].

Maqui has been selected for further investigation based on AC and TPC results. The raw material was a marketed maqui product (native, fresh, and lyophilized maqui powder). In order to evaluate the activities of maqui berry extracts as an antioxidant and anti-inflammatory potential agent, a modified procedure was implemented to develop a multigram and more efficient methodology to provide appropriated quantities. The extraction procedure described here is based on a 50 g scale for the first time, and many laboratory operations have been reduced to simplify and minimize economic costs and increase safety, since less solvents are manipulated. The reproducibility of this procedure was confirmed via all the analyses carried out of the numerous batches obtained, preserving in all cases the TPC and AC characteristics due to low temperatures and darkness along all the processes. The multigram procedure involves an improvement of the previous method published by Viuda-Martos et al. [20], in which 3 g of lyophilized and ground maqui berry were used to test antibacterial properties. Other studies submitted 1 g to solid-liquid extraction, such as in the procedure recently described by Silvia Rossi et al. [30], that uses this extract embedded in an appropriate gel for a better delivery for inflammatory bowel disease. Moreover, 1.5 g were extracted by Fredes et al. [40] to evaluate how the maturation affects the TPC. In general, all the procedures are based on the same type of extraction and all of them used an aqueous methanol acidic mixture, choosing either HCl or formic acid to accomplish a better polyphenolic extraction. Interestingly, no high temperatures above 50 °C are used in any of them, since as previously described, it might be expected that the temperature during thermal drying technologies contributed to the loss of phenols. Temperatures above 60 °C are not favorable owing to inducing oxidative condensation/decomposition of thermolabile components. Additionally, the drying method such as lyophilization preserves TPC as described by Quispe et al. [21]. It is well known that the Folin-Ciocalteu method is the most common analysis to estimate the TPC in many fruits and foods and also shows a good linear correlation to the anthocyanin content described by HPLC [44]. Although this method often overestimates the phenol content due to the presence of *L*-ascorbic acid (vitamin C) fortunately, it has been well established that maqui would not present this interference since no traces of vitamin C has been detected in maqui fruit, according to Miranda-Rottmann et al. [28]. In this sense, the TPC obtained in this study was 39.02 mg/g. Genskowsky et al. [20] described the three main groups of polyphenolic compounds in maqui berries such as phenolic acids, flavonoids, and anthocyanins, identifying a TPC of 49.49 g GAE/kg, while Reyes-Farias et al. [45] published a TPC of 19.06 mg/g, much higher values when compared with other native Chilean berries and traditional berries such as blueberries [39]. Moreover, in different maqui genotypes belonging to four different geographical regions in Chile, TPC values ranged between 11.1 and 14.5 mg/g. Brauch et al. reported that data for fresh and dry maqui berries, collected in the Aysén region (Patagonia, Chile), displaying values between 19.7 and 32.0 mg/g, were always referred to as gallic acid equivalents. On the other hand, and as previously mentioned, TPC is affected by various factors related to maqui such as genotype, environment, storage, and processing or stage at harvest. Hence, fruits with different maturity stages have different types or contents of polyphenols. Our maqui berry extract comes from the sylvester variety, which interestingly has been described as the one that contains more polyphenols if compared with the other varieties named as “Luna Nueva” and “Morena” [19]. Moreover, when the phenolic content of maqui is compared with different varieties of berries, including red wine as it is a known rich source of dietary phenols, it is possible to observe that the maqui fruit is superior, even when compared with fruits of habitual consumption, showing the maqui difference values of up to 80 times [28,45].

In addition, the AC expressed as FRAP of our maqui berry extract was determined following the method previously described. The results indicate that the maqui extract has an AC of 69.48, expressed as mmol Trolox equivalent/100 g of dried sample regarding the AC of the maqui extract to reduce Fe^3+^ to Fe^2+^. When the AC of Ach is compared with other berries, it is possible to observe significant differences, with a ferric reduction potential of 69.48 mmol Trolox/100 g compared to blueberries, which have a value of 5.9 mmol Fe^+2^/100 g dry weight [45]. Moreover, our data is much better than the other maqui extracts previously reported, that have shown FRAP values of 10.07 mmol Trolox/100 g [20]. Interestingly, maqui has several phytochemicals, particularly anthocyanins, a set of water-soluble pigments, which represent more than 65% of the total polyphenols that are responsible for the intense red to purplish-blue color of several fruits and that are influenced by pH, light or temperature. Recent studies have demonstrated that the most abundant compounds of maqui are delphinidin derivatives and cyanidin derivatives. In this sense, in the analytical UHPLC-MS/MS semi quantitative determination, the profile obtained from the maqui composition extract concerning this study was compared to the previously described study by Genskowsky et al. [20], who used a smaller extraction procedure (Figure 2). In general terms, the concentration obtained in both cases for most of the components coincide. Specifically, among the most abundant are delphinidin-3-*O*-glucoside, delphinidin-3,5-*O*-diglucoside, cyanidin-3-*O*-glucoside or delphinidin-3-*O*-sambubioside-5-*O*-glucoside. Other similar structures identified for the first time as part of a maqui extract, to our knowledge, were malvidin-3-*O*-glucoside, pelargonidin-3-*O*-glucoside, and peonidin-3-*O*-glucoside, that represent in sum 0.85% of the total composition.

The effect of polyphenolic maqui extract, related to the different mechanisms associated with inflammation and oxidation has also been described in the literature (Table 3). Once the total chemical structural determination and characterization were demonstrated, in vitro studies were carried out to investigate the biological role of these entities as potential therapeutic agents relative to OS. In this sense, it has been suggested that OS is involved in the pathogenesis and development of IBD through many levels such as cell transformation, apoptosis, DNA damage, and pro-inflammatory response [7]. Additionally, polyphenols suppress inflammation-related gene expression, downregulate proinflammatory cytokine expression, and increase the production and effects of anti-inflammatory cytokines [46].

Studies in culture cells exposed to H_2_O_2_ are a good model of OS since H_2_O_2_ is catalyzed by Fe^2+^ (Fenton reaction) to the highly reactive hydroxyl radical (HO**^●^**). However, the loss of cell viability or lipid peroxidation in epithelial cells are achieved when cells are incubated at a concentration above 250 μM H_2_O_2_ [48]. Therefore, a higher concentration of 500 μM (0.05%) for inducing oxidation and death cell in HT-29 cells was used. In the present study, we found a significant improvement in cell viability and reduction of OS when the HT-29 cells were treated with Ach, finding the main effects at doses of 300 μg/mL and at 48 h. These results indicated that the effects of Ach on HT-29 cells are both concentration and time dependent, probably due to the cellular uptake sustained over time, allowing the recovery of cell damage. These results are consistent with previous ones reported by Miranda-Rottmann et al. [28], where incubation with Ach juice prior to the addition of 500 μM H_2_O_2_ in HUVEC cells significantly reduces oxidation in a dose-dependent manner compared to endothelial cells exposed to H_2_O_2_ without treatment. Furthermore, a previous study has reported that the red wine polyphenol extract did not affect the viability of HT-29 cells at several concentrations (200, 400, and 600 μg/mL^−1^) [49], suggesting the absence of cytotoxic profile. However, higher concentrations of polyphenols such as resveratrol could induce cell death by diverse mechanisms in tumor cell lines, including colorectal cancer cells and, acting as a direct cytotoxic agent [50]. In addition, macrophages have an important role in an inflammatory response and are the central cells that initiate the production of inflammatory mediators. Macrophages stimulated with LPS, viruses, and bacterial endotoxin are able to release inflammatory factors including prostaglandin, proinflammatory cytokines, and ROS. Therefore, the regulation of the level of these factors has become vital for the treatment of inflammatory diseases [51]. In this study, we demonstrated that our extract (Ach) presented a protective activity against OS (DCF) on RAW 264.7 cells, along with the absence of cytotoxicity effect, in the same way as reported by Cespedes et al. [24]. Moreover, it has been demonstrated that other natural products decrease intracellular ROS levels in LPS-stimulated macrophages [52,53], indicating the close relationship between oxidative mechanisms and inflammation. An experimental study published by Zhou et al. [29] showed that the ethyl acetate fraction of maqui extract (MWE) contained a high content of total phenols and flavonoid and that exhibited stronger antioxidant activities than the other extracts such as *n*-butanol fraction. Additionally, the authors proved that MWE considerably reduced the expression of COX-2 and IL-6 in LPS-stimulated RAW 264.7 cells, demonstrating the anti-inflammatory effect. In addition, interestingly, in our work it was possible to show that when we used lower concentrations of Ach and a lower dose of 5-ASA the results on the percent oxidation was significantly lower compared to the control group and the other doses. This effect could indicate synergistic effects between Ach and 5-ASA. The finding of this synergy is very interesting since it opens the possibility of a combination therapy with lower doses of 5-ASA, which will lead to less side effects of this drug used in IBD patients. Furthermore, in agreement with other authors [49], we believe that the protective effect of our extract rich in total polyphenols may be due to the sum of all its components probably due to a synergic action of anthocyanins compounds suggesting that the use of total extracts in polyphenolic compounds, rather than pure polyphenols, could be a better alternative or complementary therapeutic option.

## 5. Conclusions

Our study shows that Chilean berries, specifically maqui, is a potential source of natural antioxidants and that the preservation method seems to be crucial in maintaining the AC in native berries, with fresh and frozen being the best conditions. In this study, a fresh maqui berry extract procedure has been optimized for the first time in multigram scale (50 g) via a simplified and easily reproductible laboratory procedure. The extract profile has been analyzed to quantify the TCP and AC, as well as to prove that this method fulfills the data standard previously described for smaller procedures. All these data confirm that our extraction procedure not only increases the amount of dried extract amount, but also assures an efficient process that preserves the total polyphenols, anthocyanins profile, and antioxidant properties. Interestingly, our studies in activated macrophage and epithelial colon cell cultures have shown that the maqui polyphenolic extract is a powerful antioxidant that exhibits a dose-dependent behavior and no cytotoxic effects, due to the ability to reduce ROS. In addition, our maqui extract seems to have a synergistic effect with 5-ASA, a drug of clinical use in IBD. These findings point out to a possible mechanism of action related to reduce ROS and its relationship with inflammation. However, more information concerning higher *n* values and better determination of endogenous AC will be reported by our group in due course.

In summary, maqui extracts could introduce bioactive candidates for further investigation as potential anti-inflammatory agents. Nevertheless, additional studies are necessary to analyze the effects on bioaccessibility or bioavailability of the bioactive compounds that are under current investigation in our group.

## Figures and Tables

**Table 1:** DPPH and ORAC assay results for maqui and murta extracts in three categories of preservation.

Sample	EC50 for DPPH (mg/mL)			ORAC Value (mg TE/g DM)		
	Fresh (*x* ± SD)	Refrigerated (x ± SD)	Frozen (*x* ± SD)	Fresh (*x* ± SD)	Refrigerated (*x* ± SD)	Frozen (*x* ± SD)
Maqui	372.37 ± 2.52 *	349.05 ± 1.05 *^,^^†^	364.76 ± 1.96 *	6.02 ± 0.04 *	5.93 ± 0.12 *^,†^	5.97 ± 0.56 *
Murta	79.68 ± 1.53	74.72 ± 0.58 ^†^	78.02 ± 2.04	3.97 ± 0.06	3.84 ± 0.06 ^†^	4.01 ± 0.04

Data are presented as mean ± standard deviation (SD); mg TE/g DM: Milligrams of Trolox equivalents per gram of dry mass. * Significant difference (*p* < 0.05) between berries per category of preservation. † Significant decrease (*p* < 0.05) of antioxidant capacity of maqui and murta refrigerated in comparison with fresh and frozen samples. EC50: the half maximal effective concentration; DPPH: 2,2-diphenyl-1-picrylhydrazyl; ORAC: oxygen radical absorbance capacity.

**Table 2:** Quantification values of TPC in maqui and murta by the Folin-Ciocalteu method in three categories of preservation.

Sample	Fresh (*x* ± SD) mg GAE/g FW	Refrigerated (*x* ± SD) mg GAE/g FW	Frozen (*x* ± SD) mg GAE/g FW
Maqui	75.348± 1.53 *	69.652± 0.58 *^,†^	73.786± 3.06 *
Murta	48.041± 1.00	42.780± 0.56 ^†^	47.309± 2.09

Data are presented as mean ± standard deviation (SD); mg GAE/g FW: Milligrams of gallic acid equivalent per gram of fresh weight; TCP: Total polyphenolic content. * Significant difference (*p* < 0.05) between berries per category of preservation. † Significant decrease (*p* < 0.05) of TCP of maqui and murta refrigerated in comparison with fresh and frozen samples.

**Table 3:** Anti-inflammatory and antioxidant effect of maqui berry extract described on cell cultures.

Reference	Cell Culture	Model	Extract	Concentration	Effects
Zhou G. et al. 2019 [29]	RAW 264.7 macrophage cells	Inflammatory model with LPS stimulated for 24 h	Water fraction extract with ethyl acetate rich in phenols	2–20 μg ml^−1^	↓COX-2 ↓ IL-6
Tenci M. et al. 2019 [30]	Fibroblasts and Caco-2	Oxidant model with H_2_O_2_ (1 mM) for 24 h	MBE with acid MeOH 0.1% (H_2_O:MeOH/10:90 *v*/*v*) rich in anthocyanins	MBE solution (0.5% *w*/*w*) diluted at 1:2, and 1:5 *v*/*v*	No cytotoxic effect Viability under oxidative damage
Moon HD. et al. 2019 [47]	RAW 264.7 macrophage cells	Inflammatory model with LPS stimulated for 24 h (0.1 µg/mL)	Water extract of maqui rich in anthocyanins	62.5, 125, 250, 500, 1000, and 2000 µg/ml	↓NO
Céspedes, C.L., et al. 2017 [24]	RAW 264.7 macrophage cells	Inflammatory model with LPS stimulated for 24 h (1 μg/mL)	Pulp extract with acid MeOH 0.1% HCl:H_2_O/6:4 *v*/*v*); Acetone/MeOH; Ethyl acetate	100 µg/ml	No cytotoxic effect ↓Oxidation ↓NO ↓iNOS ↓COX-2
Reyes-Farias, M., et al. 2015 [45]	RAW 264.7 macrophage cells	Inflammatory model with LPS stimulated for 24 h (5 μg/mL) or with CM from fully differentiated 3T3-L1 adipocytes	TCP: from Ripe fruits extract with acid MeOH:H_2_O/1:1 *v*/*v*)	100 μM	**CM:** ↓NO; iNOS ↑TNF-α; IL-10 **LPS:** ↓NO; iNOS; TNF-α; IL-10
Miranda-Rottmann S. et al. 2002 [28]	Primary culture of HUVEC	Vascular OS model with 500 μM H_2_O_2_	Aqueous fraction juice extract with ethyl acetate at pH 2.0 rich in anthocyanins	0.1–10 μM	↓ intracellular OS
		Copper-induced LDL oxidation in vitro		1 μM GAE	↓ LDL oxidation

(LPS): lipopolysaccharide; CM: conditioned media; OS: oxidative stress; LDL: low-density lipoprotein; MBE: maqui berry extract; TCP: total polyphenolic content; COX-2: cyclooxygenase-2; IL-6: interleukin-6; NO: nitric oxide; iNOS: inducible nitric oxide synthase; TNF-α: tumor necrosis factor-α; IL-10: interleukin-10.

## Data Availability

Not applicable.
